# Comparison of CEA and CA19-9 as a predictive factor for recurrence after curative gastrectomy in gastric cancer

**DOI:** 10.1186/s12893-022-01667-z

**Published:** 2022-06-03

**Authors:** Chikashi Shibata, Toru Nakano, Akihiro Yasumoto, Atsushi Mitamura, Kentaro Sawada, Hitoshi Ogawa, Tomoya Miura, Ichiro Ise, Kazuhiro Takami, Kuniharu Yamamoto, Yu Katayose

**Affiliations:** Division of Gastroenterologic Surgery, and Division of Hepatobiliary-Pancreatic Surgery, Department of Surgery, Tohoku Medical and Pharmaceutical University, 1-12-1 Fukumuro, Miyagino-ku, Sendai, 983-8512 Japan

**Keywords:** Carbohydrate antigen 19-9, Carcinoembryonic antigen, Gastrectomy, Gastric cancer, Tumor marker, Recurrence

## Abstract

**Background:**

Our aim of was to compare importance of the tumor markers (TMs) serum carcinoembryonic antigen (CEA) and carbohydrate antigen (CA) 19-9 in prediction of recurrence after curative gastrectomy for gastric cancer.

**Methods:**

We reviewed retrospectively the clinical records of 149 patients who underwent curative gastrectomy for stage I–III gastric cancer and whose CEA and CA19-9 levels were determined once preoperatively and for more than 3 years postoperatively. We investigated whether the clinicopathological characteristics of patients including age, sex, pathological disease stage, operative approach, type of gastrectomy, and degree of lymph node dissection as well as preoperative positivity of CEA and CA19-9 were risk factors for recurrence in univariate and multivariate analyses. Rate of recurrence was compared between patients positive and negative for postoperative CEA or CA19-9. We also calculated sensitivity, specificity, positive and negative predictable values of postoperative positivity of CEA and CA19-9 for recurrence. The lead time was compared between CEA and CA19-9 that was defined as the time of the first detection of increases in tumor markers and confirmation of recurrence on imaging modalities.

**Results:**

The number of patients positive for preoperative CEA was 25 (17%) and for CA19-9 was 11 (7%). Recurrence was confirmed in 29 (19%) patients. Stage III disease, preoperative positivity for CA19-9 but not CEA, and total gastrectomy were risk factors for recurrence in univariate analysis, but stage III disease was the only risk factor for recurrence in multivariate analysis. Forty and 15 patients were positive for postoperative CEA and CA19-9, respectively. The recurrence rate of 47% (7/15) in patients positive for postoperative CA19-9 was greater than that in negative patients (22/134 = 16%), but it did not differ between patients who were positive or negative for postoperative CEA. Specificity for CA19-9 was greater than that for CEA (P < 0.05). The lead time of CEA (3.9 ± 4.7 months) was not different from that of CA19-9 (6.1 ± 7.1 months).

**Conclusions:**

These results indicate that CA19-9 rather than CEA is likely to be more useful for the detection of recurrence after curative gastrectomy for gastric cancer.

## Introduction

Gastric cancer has a high prevalence in East Asia, and is the 3rd most common cause of death due to malignant diseases in Japan [1]. Although the prognosis in patients with stage I gastric cancer is excellent, 30–60% disease free survival for patients with stage III indicates that considerable number of patients recur despite a “curative” gastrectomy [2, 3]. Because early detection/intervention for recurrent gastric cancer after a curative gastrectomy is important [4, 5], prediction of recurrence with measurement of serum levels of tumor markers (TMs), which is a non-invasive and relatively cheap, would be of great importance for such patients.

Carcinoembryonic antigen (CEA), carbohydrate antigen (CA) 19-9, alfa-fetoprotein, CA72-4, and CA125 are representative TMs for gastric cancer and considered useful in detecting the recurrence after curative gastrectomy [6]. Among these TMs, CEA and CA19-9 are the most classic TMs [7, 8] and are measured regularly after gastrectomy in the daily clinical practice in many institutions, including ours. CEACAM6 (CEA-related cell adhesion molecule 6) is a cell adhesion protein of the CEA family and expressed on normal epithelial tissue, and its overexpression is associated with development, invasion, and metastasis of cancer [9]. Blockade of CEACAM6 reactivated the antitumor response of T cells in a previous study using cell lines [10], thus CEACAM6 might have something to do with angiogenesis and immunosuppression which is important in the growth of cancer [11]. CA19-9 is often increased in patients with pancreatic ductal adenocarcinoma as well as other gastrointestinal cancer including gastric cancer, and expression CA19-9 is associated with hyperactivation of epidermal growth factor receptor [12].

Many previous reports evaluated preoperative but not postoperative serum levels of TMs as a risk factor for recurrence [13–17]. Serum levels of TMs are measured sequentially more than once at certain intervals postoperatively. Few reports, however, have investigated the relationship between postoperative positivity of TMs and recurrence [18, 19].

The aim of the present study was to compare the efficacy of CEA and CA19-9 in prediction of recurrence after curative gastrectomy for gastric cancer. We analyzed patients in whom CEA and CA19-9 levels were measured both preoperatively and postoperatively after a curative gastrectomy.

## Patients and methods

This study was approved by our institutional review board (2020-2-143). We reviewed retrospectively the clinical records of the patients who underwent a curative distal gastrectomy (DG), proximal gastrectomy (PG), or total gastrectomy (TG) for stage I–III gastric cancer between 2009 and 2016. Patients with remnant gastric cancer were excluded, as were those who underwent non-curative resection, those who had a malignant disease other than gastric cancer, and those who had been treated with neoadjuvant chemotherapy for gastric cancer. Among those patients who fulfilled the above criteria, patients were included in this study whose CEA and CA19-9 levels were determined at least once within 1 month before their gastrectomy, and at 3–6 month intervals postoperatively for more than 3 years or until the confirmation of recurrence on imaging modalities. Preoperative CEA and CA19-9 levels greater than 5 ng/mL and 37 U/mL respectively were regarded as positive [18, 19]. We defined cut-off level of ‘slight’ increase for CEA levels less than 10 ng/mL and CA19-9 levels less than 60 U/mL based on previous reports [19–21]. Regarding the postoperative analysis, patients were judged to be positive when their serum levels of CEA and CA19-9 exceeded the normal range even once before confirmation of recurrence on imaging modalities. Recurrence was diagnosed on imaging modalities, including chest X-ray, computed tomography scan, magnetic resonance imaging, and abdominal ultrasonography.

First, we performed univariate and multivariate analyses to determine if perioperative clinicopathologic characteristics (patients age at the time of operation, sex, pathological disease stage, operative approach, type of gastrectomy, and the degree of lymph node (LN) dissection) as well as whether preoperative CEA or CA19-9 positivity were risk factors for recurrence. The pathologic stage of the gastric cancer and the degree of LN dissection (D1, D1+, D2) were classified based on the Japanese classification of gastric carcinoma and the Gastric Cancer Treatment Guidelines in Japan [22, 23].

Regarding postoperative positivity of CEA and CA19-9, the association between recurrence and pre- and postoperative positivity was investigated using a cross tabulation table, and we compared recurrence rate between postoperatively positive and negative patients for CEA and CA19-9. We also studied the sensitivity, specificity, positive predictable value (PPV), and negative predictable value (NPV) of postoperative positivity of CEA and CA19-9 for recurrence. The lead time was compared between CEA and CA19-9, which was defined as the time of the first detection of increased TMs postoperatively subtracted from the time of the recurrence diagnosed with imaging modalities [24].

Fisher’s exact probability test and logistic regression analysis were performed for univariate and multivariate analyses, respectively. We used Mann–Whitney U test for comparison of the lead time. Chi-square test was used for other comparisons. Values are shown in mean ± standard deviation, and P values less than 0.05 were regarded as significant.

## Results

### Preoperative positivity of CEA and CA19-9 and recurrence

Clinical characteristics of the 149 patients are shown in Table 1. Mean follow-up time was 3.9 ± 1.4 years. Median age was 67 years, and 70% of the patients were male. Thirty-two percent of the patients were stage III. The number of patients positive for preoperative TMs was 25 (17%) for CEA, and 11 (7%) for CA19-9. Open surgery was performed in 79% of the patients, and 35% of whom underwent TG. Lymph node dissection was D1 and D1+ in 57% of the patients. Adjuvant chemotherapy with S-1, an orally administrable 5-fluorouracil agent, was performed in 33% of the patients. There were 29 (19%) patients with recurrences, and sites of recurrence with some duplications were peritoneum in 13, lymph node in 10, liver in 7, bone in 2, abdominal wall in 2, and pleura and pancreas in 1, respectively.

**Table 1:** Characteristics of 149 patients

Median age (Range)	67 (41–87)
Sex	
Male	105 (70)
Female	44 (30)
Disease stage	
I, II	102 (68)
III	47 (32)
Preoperative CEA	
Positive	25(17)
Negative	124 (83)
Preoperative CA19-9	
Positive	11 (7)
Negative	138 (93)
Approach of operation	
Open	118 (79)
Laparoscopic	31 (21)
Type of gastrectomy	
DG, PG	97 (65)
TG	52 (35)
Lymph node dissection^a^	
D1, D1+	85 (57)
D2	64 (43)
Adjuvant chemotherapy	
Yes	49 (33)
No	100 (67)
Recurrence	
Patients with recurrence	29 (19)
Patients without recurrence	120 (81)

Numbers in parentheses are %

^a^Classification according to the Japanese classification of gastric carcinoma and the Gastric Cancer Treatment Guidelines in Japan

Table 2 summarizes results of univariate analysis in terms of recurrence and the 8 perioperative factors. The rate of stage III, preoperative positivity for CA19-9, and TG in patients with recurrence was greater than that in patients without recurrence in univariate analysis. All other factors did not relate to recurrence including age, sex, preoperative CEA positivity, operative approach, and extent of LN dissection. The eight factors above were investigated in multivariate analysis, with disease stage being the only independent risk factor for recurrence (Table 3). Rate of patients positive for preoperative CA19-9 in stage III was 15% (7 of 47 patients), and this value was greater than that in stages I and II as 4% (4 of 102 patients: P < 0.05). While regarding preoperative CEA, rate of positive patients did not differ between stage III (10/47, 21%) and stages I and II (15/102, 15%).

**Table 2:** Results of univariate analysis

	Patients with recurrence (N = 29)	Patients without recurrence (N = 120)	P value
Age			
≥ 65	20 (69)	68 (57)	0.2938
< 65	9 (31)	52 (43)	
Sex			
Male	18 (62)	87 (73)	0.2676
Female	11 (38)	33 (27)	
Disease stage			
I, II	5 (17)	97 (81)	< 0.0001
III	24 (83)	23 (19)	
Preoperative CEA			
Positive	4 (14)	21 (18)	0.7854
Negative	25 (86)	99 (82)	
Preoperative CA19-9			
Positive	6 (21)	5 (4)	0.0076
Negative	23 (79)	115 (96)	
Operative approach			
Open	25 (86)	93 (78)	0.4446
Laparoscopic	4 (14)	27 (22)	
Type of gastrectomy			
DG, PG	13 (45)	84 (70)	0.0162
TG	16 (55)	36 (30)	
LN dissection			
D1, D1+	15 (52)	70 (58)	0.5373
D2	14 (48)	50 (42)	

Numbers in parentheses are %

**Table 3:** Results of multivariate analysis

	Odds ratio	95% CI	P value
Age ≥ 65	1.10	0.37–3.31	0.863
Gender	0.66	0.22–1.93	0.445
Disease stage	0.04	0.01–0.16	< 0.01
Preoperative CEA	1.69	0.43–6.65	0.455
Preoperative CA19-9	0.25	0.05–1.31	0.102
Approach of operation	1.42	0.30–6.76	0.660
Type of gastrectomy	0.47	0.14–1.57	0.217
LN dissection	0.68	0.19–2.40	0.544

*CI* confidence interval

We investigated TM levels in detail in patients positive for preoperative TMs. Patients with slightly increased levels (less than 10 ng/mL) occurred in 68% (17 of 25) of patients positive for preoperative CEA, and 1 of these 17 patients developed recurrence. In the 11 patients positive for preoperative CA19-9 levels, there was only 1 (9%) patient with a slight increase (less than 60 U/mL), and the recurrence was not observed in this patient. Conversely, 3 of 8 (38%) whose preoperative CEA was > 10 ng/mL and 6 of 10 (60%) whose CA19-9 was > 60 U/mL developed recurrence.

### Postoperative positivity of CEA and CA19-9 and recurrence

Table 4 is a cross tabulation table showing number of patients with recurrence in association with preoperative/postoperative positivity for CEA and CA19-9. Among 25 patients positive for preoperative CEA, 20 (80%) of these remained positive postoperatively, and the recurrence was identified in 3 of these 20 (15%) patients. There were 11 patients positive for preoperative CA19-9, and 9 of these 11 (82%) patients were also positive postoperatively. The recurrence was observed in 5 of these 9 (56%) patients, and this percentage was greater than that of CEA (56% vs. 15%: P < 0.05). There were overall 40 and 15 patients positive for postoperative CEA and CA19-9, respectively (Table 4). Recurrence was observed in 10 of 40 patients (25%) positive for postoperative CEA; importantly, this value did not differ from recurrence rate of 17% (19/109) in patients negative for postoperative CEA. The recurrence rate of 47% (7/15) in patients positive for postoperative CA19-9 was greater than that in negative patients (22/134 (16%), Table 4: P < 0.05). The lead time of CEA (3.9 ± 4.7 months) was not different from that of CA19-9 (6.1 ± 7.1 months). Specificity for CA19-9 (112/120 (93%)) was greater than that for CEA (90/120 (75%), P < 0.05). Sensitivity, PPV, and NPV for CEA were 10/29 (34%), 10/40 (40%), and 90/109 (83%), respectively; these values did not differ from those for CA19-9 (7/29 (24%), 7/15 (47%), and 112/134 (84%)).

**Table 4:** Number of patients with recurrence in association with preoperative/postoperative positivity for CEA and CA19-9

	Postoperative positivity		
	Negative	Positive	Total
CEA			
Preoperative positivity			
Negative	18/104 (17)	7/20 (35)	25/124 (20)
Positive	1/5 (20)	3/20 (15)	4/25 (16)
Total	19/109 (17)	10/40 (25)	29/149 (19)
CA19-9			
Preoperative positivity			
Negative	21/132 (16)	2/6 (33)	23/138 (17)
Positive	1/2 (50)	5/9 (56)	6/11 (55)
Total	22/134 (16)	7/15 (47)	29/149 (19)

Numbers in parentheses are %

## Discussion

In the present study, we investigated if preoperative positivity of CEA and CA19-9 were risk factors for recurrence in univariate and multivariate analyses. We used different methods of analysis to evaluate postoperative positivity of CEA and CA19-9, with consideration that the temporal measurements of TMs were carried out more than once postoperatively. As a result, we found that stage III disease, undergoing a TG, and preoperative positivity for CA19-9 but not CEA were risk factors for recurrence in univariate analysis. In multivariate analysis, stage III disease was the only risk factor for recurrence. We also found that rate of preoperative positivity for CA19-9 in stage III patients was greater than that in stages I and II patients, but the same phenomenon was not observed for CEA. The recurrence rate in patients positive for preoperative and postoperative CA19-9 was greater than that for CEA. The recurrence rate in patients positive for postoperative CA19-9 was greater than in negative patients, but it did not differ between patients who were positive and negative for postoperative CEA. Sensitivity for recurrence of postoperative positivity of CA19-9 was greater than that of CEA. These results suggest the possibility that CA19-9 rather than CEA is of more help in surveillance and prediction of recurrence after curative gastrectomy. The observation that the lead time for CA19-9 tended to be about 1.5 times greater than that for CEA does not conflict with our conclusion, although the difference was not statistically significant.

A previous study suggested that preoperative serum CA19-9 was better than CEA as a prognostic factor in multivariate analysis [13]. There was a difference in 5-year survival in stages II, III, and IV disease between patients with high vs. low preoperative serum CA19-9, but not for CEA [14]. Preoperative serum levels of CA19-9 but not CEA were useful as a prognostic factor, but patients with positive CEA values had a significantly high risk of lymph node metastases [15]. These results in previous studies support our findings that CA19-9 is likely to be more useful than CEA as a predictive factor for recurrence. In contrast, several previous reports appear to be in conflict with ours; two studies concluded that multivariate analysis did not identify preoperative positivity of CA19-9 as statistically significant prognostic factors [25, 26], while the prognostic values of preoperative CEA levels were confirmed in several studies [16–20]. Thus, there have been few studies that analyzed levels of CEA and CA19-9 both pre- and post-operatively like in the present study, we believe that our results include new information on the relationship between levels of TMs and recurrence.

The increase in CEA levels was slight (less than 10 ng/mL) in 17 of 25 patients (68%) who were positive for preoperative CEA, and only 1 patient had recurrence in these 17 patients. We consider that this result must have something to do with the fact that preoperative positivity for CEA was not a risk factor for recurrence in univariate analysis. Smoking is the most representative cause of increase in serum CEA [27, 28] other than cancer, and it is likely that ‘false positive’ patients associated with smoking were included in these 17 patients with a slight increase of preoperative CEA. We could not study past history of smoking in patients with increased preoperative CEA, because history of smoking was not described in all our clinical records, and lack of potentially relevant data is a limitation of this study.

A phenomenon has often been observed that the increase in TMs precedes the confirmation of recurrence on imaging modalities, and this is considered one of the important to measure serum TMs postoperatively. However, it is very likely that the improvement of imaging technology influences the lead time; the lead time for CEA was 8.3 months in 1982, and it was as short as 3–5 months after 2000 [6, 24, 29]. This shortened lead time in recent years must be due to development of more sensitive and specific imaging modalities. We found that the lead time did not differ between CEA and CA19-9 as reported previously, and the values of the lead time of 3.9 months for CEA and 6.1 months for CA19-9 in ours study were comparable to those in previous reports [24, 30].

The positive rates for TMs were 7% and 10% for pre-and postoperative CA19-9, respectively, and 17% and 27% for pre- and postoperative CEA, respectively, in our study. These values are generally low compared to previous studies that showed that positivity for pre- and postoperative CA19-9 was 10–29% and 8–30% and for CEA was 13–28% and 11–33%, respectively [18, 24, 31–33]. This difference was considered associated with the difference in distribution of patient disease stage; patients with stage III disease in our study was 32%, while rate of patients with stage III disease in previous studies was greater than our study. The rate of TM positivity increases as disease stage advances [6].

We defined inclusion criteria of at least a 3-year follow up period except for patients with recurrent disease within this initial 3 year period. Therefore, it is possible that patients who may have recurred after our follow-up period were included in a group without recurrence in this study. We, however, consider that those patients are very few, because approximately 90% of gastric cancer recurrences are observed within 3 years after curative gastrectomy [34, 35]. Another limitation is that this is a retrospective study in one institution with a relatively small number of patients with recurrence.

## Conclusions

Positivity of preoperative CA19-9, but not CEA, appears to be a risk factors for recurrence in univariate analysis, although these two factors were not risk factors for recurrence in multivariate analysis. The rate of recurrence was greater in patients positive for postoperative CA19-9 levels than in patients who were negative, but it did not differ between patients who were positive and negative for postoperative CEA. Specificity for recurrence were greater for postoperative CA19-9 than CEA. These results indicate that CA19-9 rather than CEA is likely to be of more help in the prediction of recurrence after curative gastrectomy for gastric cancer.

## Data Availability

The datasets generated and/or analyzed during the current study are available from the corresponding author on reasonable request.

## Abbreviations

CA19-9: Carbohydrate antigen 19-9
CEA: Carcinoembryonic antigen
DG: Distal gastrectomy
PG: Proximal gastrectomy
TG: Total gastrectomy
